# Learning From Consultations Conducted by Community Pharmacists in Northern Ireland for Nonprescription Sildenafil: A Qualitative Study Using the Theoretical Domains Framework

**DOI:** 10.1016/j.esxm.2021.100440

**Published:** 2021-10-07

**Authors:** Rineke Gordijn, Martina Teichert, Melianthe P.J. Nicolai, Henk W. Elzevier, Henk-Jan Guchelaar, Carmel M. Hughes

**Affiliations:** 1 Leiden University Medical Center, Department of Clinical Pharmacy & Toxicology, Leiden, The Netherlands; 2 Netherlands Cancer Institute-Antoni van Leeuwenhoek Hospital, Department of Urology, Amsterdam, The Netherlands; 3 Leiden University Medical Center, Department of Urology and Department of Medical Decision Making, Leiden, The Netherlands; 4 Primary Care Research Group, School of Pharmacy, Queen's University Belfast, Belfast, Northern Ireland

**Keywords:** Community Pharmacy Services, Nonprescription Drugs, Sildenafil, Theoretical Domains Framework

## Abstract

**Introduction:**

Nonprescription sildenafil was introduced to the United Kingdom in 2018 as the first pharmacy service concerning sexual function, an important but often ignored factor for quality of life.

**Aim:**

This study aimed to evaluate pharmacists’ views on providing nonprescription sildenafil, their perceptions of the barriers and facilitators to provide this service and strategies to overcome potential barriers, using a theory-based approach.

**Methods:**

Community pharmacists were purposefully sampled in Northern Ireland, followed by snowball sampling. Face-to-face interviews were conducted between October 2019 and January 2020. The semi-structured interviews used a piloted topic guide based on the 14-domain Theoretical Domains Framework (TDF). All interviews were audio-recorded, transcribed verbatim and anonymized. Transcripts were analyzed deductively in NVivo 13, utilizing the TDF domains as coding categories. Within each domain, content analysis was utilized to identify barriers and facilitators.

**Main Outcome Measure:**

Barriers and facilitators within the TDF domains for pharmacists to provide nonprescription sildenafil.

**Results:**

Ten pharmacists were interviewed to reach data saturation. Eight pharmacists had experience with dispensing nonprescription sildenafil. They valued nonprescription sildenafil as an additional service (“Social/professional role and identity”). Training, concise product guidelines, and private consultation areas were important facilitators (“Environmental context and resources”). The service required trusting clients (“Optimism”), with concerns about abuse and men not visiting their GP. From experience gained, pharmacists became more confident dealing with difficult situations such as patients being vague about their medical history or alcoholism or mental problems as causes for erectile disfunction (ED) (“Skills” and “Beliefs about capabilities”). Pharmacists considered lifestyle and medication causes of ED important but preferred to focus on safe supply. In general, pharmacists were satisfied with the perceived professional recognition, using their clinical knowledge or helping patients resume sexual relationships (“Beliefs about consequences”).

**Conclusion:**

Pharmacists welcomed nonprescription sildenafil to enhance their role as easily accessible healthcare providers for patients.

**Gordijn R, Teichert M, Nicolai MPJ, et al. Learning From Consultations Conducted by Community Pharmacists in Northern Ireland for Nonprescription Sildenafil: A Qualitative Study Using the Theoretical Domains Framework. Sex Med 2021;9:100440.**

## INTRODUCTION

A broad range of diseases and treatments can lower sexual functioning, which many patients consider an essential part of their quality of life.^1^ The importance of sexuality is acknowledged by persons of all ages, including those with medical conditions and those who are older.^2^, ^3^, ^4^, ^5^ For instance, patients with schizophrenia expressed that the subjective burden of sexual dysfunction can be as high as the burden of the disease itself.^6^ Patients on antidepressants reported sexual adverse drug reactions (sADRs) as some of the most difficult side effects to live with.^7^ Moreover, sexual problems can significantly impact individuals’ mental health and relationships, consequently being described as the most disturbing part of living with diabetes.^8^^,^^9^

Although patients and healthcare providers acknowledge the importance of sexuality, it is not routinely addressed in healthcare. Since the International Conference on Population and Development (ICPD) included sexual health in the definition of reproductive health in 1994, sexuality has slowly become part of healthcare policy, for example, being reported in disease-specific guidelines and with new legislation approving emergency contraception as over-the-counter (OTC) medication.^10^^,^^11^ Nonetheless, such developments have not yet progressed to sexual functioning being routinely addressed by healthcare professionals.^12^, ^13^, ^14^, ^15^, ^16^ Barriers have been reported, such as lack of knowledge, lack of time, worrying about causing offence, and assuming low priority.^12^, ^13^, ^14^^,^^16^^,^^17^ Additionally, healthcare professionals assumed that patients would seek help when needed. However, only 1 in 4 men and 1 in 5 women with sexual complaints indeed sought help,^18^ with barriers such as embarrassment, lack of awareness, and other priorities during the consultation being reported.^8^^,^^9^^,^^18^

To our knowledge, pharmacists have not been included in any research on discussing sexuality in healthcare practice. Yet pharmacists are considered well-positioned to promote health and wellbeing because of their accessibility.^19^ No appointment is needed in the community pharmacy to talk to a healthcare professional. Therefore, patients might discuss sexual health topics more readily with pharmacists. Consequently, pharmacists could be more involved in the detection and referral of patients with sexual dysfunction as it can be indicative of cardiovascular disease and depression.^20^^,^^21^ Furthermore, pharmacists could inform patients on possible sADRs. One study reported that 44% of users of oral anticancer drugs wished to receive this information.^22^

In March 2018, over-the-counter Viagra Connect (sildenafil) was introduced in community pharmacy practice in the UK, see Table 1.^23^ This service comprises pharmacists assessing if the man meets certain supply criteria (eg, indication, interactions and contra-indications) and provides further information on sildenafil and causes of erectile dysfunction (ED).

**Table 1:** Overview of OTC sildenafil service in the UK

Date of introduction	Legislation: November, 2017 Introduced in pharmacy: March, 2018
Drug and brand	Sildenafil 50mg (Viagra Connect)
Classification restrictions	For oral use
	For men with erectile dysfunction
	For adult men
	Strength: 50 mg sildenafil
	Maximum pack size: 8 tablets
	Maximum daily dose: 50 mg
Training	Recommended
	From the manufacturer online available at company's website. Regional and national training programs also available
Reasons for not supplying sildenafil and referral to GP	Cardiovascular health insufficient for sexual activity
	Patient has heart conditions or recently had a heart attack or stroke
	Patient uses concomitant medication with interaction potential
	Patient has certain concomitant diseases (eg, penile deformation, hepatic diseases)
Checklist	The manufacturer's checklist for the pharmacist consists of a 2-sided document: The first page explains the indication for Viagra Connect®, lists the contra-indications and drug interactions and gives potential causes of ED in an additional advice box The second page gives consultation points about the use of sildenafil At the bottom of the checklist are 2 tear-off slips, one for resupply and one for GP referral
Lifestyle advice	Pharmacists are recommended to provide lifestyle advice (weight, smoking, alcohol/drugs, exercise, stress)
Contact with GP	Pharmacist should recommend a doctor's visit within 6 months. Patients who cannot be supplied OTC sildenafil receive a tear-off slip from the checklist for discrete referral to the GP.
Resupply	With tear-off slip from the checklist, signed by the pharmacist who did the initial supply
	Pharmacists are recommended to ask about changes in health and medication
Repeat of consultation	Only if factors in health or medication have changed
Documentation	None required as supply is anonymous

OTC = over-the-counter; ED = erectile dysfunction; GP = general practitioner.

To understand the impact of this new service, pharmacists’ perspectives and experiences should be assessed. Because limited information is available on the perspective of pharmacists on this service, a more explorative, qualitative approach was chosen to answer questions about the “what,” “how,” and “why” of this service.^24^ Furthermore, to explore healthcare professionals’ perspectives on behavior changes related to practice, theory-based frameworks are increasingly used as they can capture a broad understanding of how a new service is integrated into practice.^25^, ^26^, ^27^

The aim of this study was to evaluate the perceptions of community pharmacists on providing nonprescription sildenafil, using a theory-based framework. The key objectives were (i) to capture the experiences of community pharmacists providing OTC sildenafil, (ii) to identify the facilitators and barriers for conducting the new pharmacy service and (iii) to explore the strategies to overcome these barriers.

## METHODS

### Study Design

This study was set within the Medical Research Council (MRC) Framework for the development and evaluation of complex interventions, focusing on the evaluation phase.^28^ To evaluate the provision and what had influenced the implementation of the nonprescription sildenafil service in community pharmacies, semi-structured interviews were conducted with pharmacists in Northern Ireland. The MRC Framework recommends the selection of a theoretical framework to identify barriers and facilitators for implementing new interventions. To meet to our objectives, the Theoretical Domains Framework (TDF) was chosen and we referred to published guidance on its use.^29^

### Theoretical Domains Framework

The TDF was developed in response to calls for explicit use of theory in intervention development and implementation, based on 33 theories of behavior and behavior change.^29^ These theories are clustered in domains which might influence the behavior of healthcare professionals. In this way, the TDF can provide a “theoretical lens through which to view the cognitive, affective, social and environmental influences on behaviour.”^29^ In pharmacy practice, the TDF has become a widely used framework to understand the behavior of the pharmacist.^25^^,^^27^^,^^30^^,^^31^ For this study, the most recent version of the TDF was used, which consists of 14 domains: knowledge; skills; social/professional role and identity; beliefs about capabilities; optimism; beliefs about consequences; reinforcement; intentions; goals; memory, attention and decision processes; environmental context and resources; social influences; emotions; and behavioral regulation.^29^

### Target Behavior

For optimal use of the TDF, Atkins et al emphasized the specification of the target behavior, that is, the behavior that needs to be changed to address the implementation problem.^29^ The target behavior for this study was specified as community pharmacists providing nonprescription sildenafil to eligible clients.

### Procedures

#### Development of topic guide

To undertake the interviews, R.G. and C.M.H. developed the topic guide based on the TDF domains, and with reference to other TDF-based studies.^27^^,^^32^ Furthermore, the topic guide was informed by a literature review, a systematic review by Dyer et al*,*^17^ and promotional and educational material provided by the manufacturer of Viagra Connect to assist pharmacists in delivering the service.

The topic guide started with an introduction about research on sADRs and the rationale for this study, followed by a small number of demographic questions. The guide then encouraged the pharmacists to think about specific nonprescription sildenafil consultations they had provided, after which they were asked to describe how the service was provided in each pharmacy. A series of TDF questions explored the various domains. The topic guides were piloted with 4 research students and research fellows who practised or had practised as community pharmacists in Northern Ireland, which facilitated refinement of the content.

#### Recruitment of community pharmacists

The sampling frame for the study was pharmacists working in community pharmacies in the greater Belfast area. Community pharmacists were initially sampled on a purposive basis, utilizing key informants known to the Northern Ireland researcher (C.M.H.). This was followed by snowball sampling. With this method it was anticipated that pharmacists with different demographic characteristics (eg, age, gender, location and type of pharmacy, if they provided nonprescription sildenafil) would be recruited. Pharmacists were invited via an invitation letter sent by email, together with an information sheet, providing further details of the study. After a few days, a follow-up email or telephone call was made to these pharmacists to ascertain interest in the study. If pharmacists were interested in participating, arrangements were made to undertake an interview. When a pharmacist had not provided the nonprescription sildenafil service, the questions from the topic guide were asked hypothetically on how the service would have been provided. Prior to the start of the study, no specific recruitment target was stated as sampling would be guided by data saturation having been achieved in relation to barriers and facilitators.^29^

Of the 13 community pharmacists who were initially approached for this study, 2 pharmacists could not be reached by telephone or email and 1 pharmacist declined because of a busy schedule. In total, 10 pharmacists participated in the study. Table 2 shows the demographic characteristics of the participants. Eight of the 10 pharmacists had dispensed OTC sildenafil, of whom 7 started as soon as OTC sildenafil became available in Northern Ireland. Two pharmacists had never provided OTC sildenafil. One of these pharmacists worked as a locum in a pharmacy where OTC sildenafil was available but never requested. The other pharmacist worked on the outskirts of Belfast and did not want to offer OTC sildenafil because of anticipated low demand, the high price in comparison to obtaining sildenafil on prescription and negative experiences with the manufacturer. A third pharmacist worked as a locum mainly in 2 pharmacies, of which 1 pharmacy did not provide OTC sildenafil. He suggested that the strong religious beliefs of the pharmacy's clientele accounted for the manager's main reason for nonprovision.

**Table 2:** Summary of the demographic characteristics of pharmacists interviewed

Pharmacist code	Gender (F = female, M = male)	Area in belfast	Employment	Type of pharmacy	Years of professional experience	Provided OTC sildenafil	Number of requests of OTC sildenafil
PH1	F	Belfast city	Owner	Independent	25	Yes	1/week
PH2	M	Belfast city	Owner	Small chain	34	Yes	1-2/week
PH3	M	Greater Belfast area	Owner	Independent	32	No	3/year
PH4	M	Greater Belfast area	Owner	Independent	20	Yes	2–3/week
PH5	M	Belfast touristic center	Owner	Independent	30	Yes	7-10/week
PH6	M	Town outside Belfast	Locum	Small chain	10	No	None
PH7	F	Belfast city	Employee	Independent	30	Yes	1/week
PH8	F	Belfast city	Locum	Large chain	2	Yes	1/shift
PH9	M	Town outside Belfast	Locum	Independent	2	Yes	1/shift
PH10	M	Belfast city and greater Belfast area	Locum	Two independent pharmacies	2,5	Yes	1-2/shift

Independent pharmacy = not associated with a chain of pharmacies; small chain = less than 5 pharmacies; large chain = 5 or more pharmacies; OTC = over-the-counter.

#### Data collection

Data was collected during face-to-face interviews at the pharmacists’ workplaces. The participants were not known to the researcher (who was Dutch [R.G.]) before the start of the study. The 10 pharmacists who agreed to participate were interviewed between October 2019 and January 2020. The interviews lasted on average 48 minutes, ranging between 28 and 65 minutes. Interviews were audio-recorded using digital recording equipment, with written consent from the participants. Each interview was transcribed verbatim by the first author, before being anonymized and entered into NVivo 13 (QSR International Pty Ltd. Version 13, 2020).

#### Data analysis

For this study and in order to describe the barriers and facilitators in providing nonprescription sildenafil, deductive analysis was chosen. The deductive analysis described by Atkins et al started with a familiarization phase in which the transcripts were read, and a general description of pharmacists’ perspectives was summarized.^29^ During this phase, the responses were initially attributed to 1 or more TDF domains, which constituted the codes for this deductive analysis.^29^ To assure reliability, the coding scheme for the attribution of domains to certain phrases was agreed during 2 consensus meetings. The first consensus was reached by 2 researchers (R.G., C.M.H.) after independently coding 3 interviews. A second consensus was reached by 3 researchers (R.G., C.M.H., M.T.) after independently coding 5 interviews. The codes were ordered in a matrix, also known as the “spreadsheet” approach.^33^ In this approach, the output is a matrix in which the rows are the participants of the interview, the columns the codes (ie, the TDF domains) and the cells summarize the data. In this way, the most important domains could be determined from the matrix, largely based on a relatively high frequency of barriers or facilitators reported by the participants.^29^ Lastly, critical appraisal of participants’ responses was undertaken to identify other issues which may not have already been highlighted.

### Ethical Considerations

Ethical approval was granted by the School of Pharmacy Ethics Committee, Queen's University, Belfast.

## RESULTS

Most pharmacists reported receiving 1–2 requests for OTC sildenafil a week, with a trend towards more requests on the weekends and in pharmacies located in the center of Belfast. Most requests were from clients who were not known to the pharmacist. The initial consultation would take between a couple of minutes to 10–15 minutes. Most pharmacists saw a growing number of resupply slips, with some reporting that they constituted between 10% and 25% of the requests and the majority signed by a different pharmacy. In 3 pharmacies, some men had become regular clients for OTC sildenafil. Most of the pharmacists had infrequently referred patients to their general practitioner (GP) without supply.

For the pharmacists, OTC sildenafil was another service in a range of services they provided and a normal part of practice. One pharmacist, whose pharmacy practice consisted of a significant OTC service, had increased the manufacturer's suggested price because he felt his time was worth more than the financial profit of selling OTC sildenafil, especially in comparison to other OTC medication.

Some pharmacists had been initially surprised that also young men requested OTC sildenafil. For the consultation, the checklist suggests providing advice on ED causes, a topic which was not addressed when men seemed uncomfortable discussing their ED problems. The pharmacists already selectively focused on a few ED causes from the checklist such as depression, anxiety, alcohol or the use of betablockers or SSRIs. However, if patients seemed unwilling to talk, most pharmacists would not start the conversation about potential ED causes. Medication-induced ED was viewed differently, as pharmacists considered themselves responsible for informing clients about sADRs. However, their opinions differed on if and how sADRs should be incorporated into the client encounter. Overall, pharmacists recognized a lack of knowledge on which drugs would impact sexual functioning. Two younger pharmacists attributed this to that fact that they had not learned about sADRs during their education.

### Facilitators and Barriers

Table 3 shows the facilitators and barriers per TDF domain. The most reported facilitators were training, the “comprehensive and concise” checklist of the manufacturer and the presence of a consultation room or private area (“Environmental context and resources”). The most common form of training undertaken was the online programme provided by the manufacturer. The knowledge provided by different modes of training was generally considered comprehensive (“Knowledge”). Some pharmacists found role plays or examples within the training particularly useful to develop the skills needed to provide nonprescription sildenafil; others stated that these skills were best learned in clinical practice (“Skills”).

**Table 3:** Facilitators and barriers experienced by pharmacists providing nonprescription sildenafil

TDF domain	Facilitators	Barriers
*Knowledge*	Training with information about the condition and the restrictions for supply	Lack of knowledge on erectile dysfunction, possible causes for the erectile dysfunction and specific interactions or contra-indications
	Checklist with the restrictions for supply	
	Understanding of the situation for men with ED	
*Skills*	Communication skills (eg, empathy, discretion, tact)	
	Negotiation skills (encouraging men to go to their GP when necessary)	
	Developing experience based on previous consultations or from receiving skills training with role plays or cases	
*Social/ Professional role and identity*	Service adding to recognition that pharmacist is a healthcare professional	Patients' expectation that they can buy nonprescription sildenafil without consultation
	Clear and reasonable supply restrictions	
	Using the service as a way to improve public health	Men talking about their sex life
*Beliefs about capabilities*	Experience from previous consultations helping to build confidence	Language or cultural barriers impeding communication
	Knowing the patient or knowing the ED cause	Insufficient information provided by clients
	Being confident that the consultation went well because patients were happy to talk, were healthy or who clearly reported red flags (eg, contra-indications) for referral	Men with no clear ED cause or the cause being alcoholism or psychological problems
		Men not understanding the reason for a consultation and/or unwilling to engage with the consultation
*Optimism*	Patients coming back for re-supply	Having to trust the patients’ answers
	Increased requests for nonprescription sildenafil	Concern that referred patients will not go to the GP or will seek a supply in another pharmacy
	No signs of inappropriate supplies, complaints from clients or public criticism	
*Beliefs about consequences*	Being able to show the potential of pharmacists as healthcare professionals	Fear of receiving inappropriate requests (eg, not to treat ED, but for better performance)
	Using the service as a way to improve public health	
	Developing a professional relationship with men who return for re-supply	High price of nonprescription sildenafil in comparison to prescription sildenafil
	Belief that the service benefits the client, pharmacy profession, individual pharmacy and healthcare system	
*Reinforcement*	Patients coming back for re-supply	Patients not returning for re-supply
	Receiving requests from patients who the pharmacist knows, signifying a trusted relationship	Low demand for nonprescription sildenafil
	Receiving a financial benefit from selling OTC sildenafil	Clients misusing the service
	Satisfaction with professional recognition of the pharmacist because of this new service	Embarrassing consultations
	Using the service as a way to improve public health	
	Perception of helping a client overcome relationship difficulties that were (partly) caused by erectile dysfunction	
*Intentions*	Commitment to being part of development of the professional role	Commitment to only providing nonprescription sildenafil to clients known to the pharmacist
*Goals*	Wanting to contribute to the development of the professional pharmacy role	
*Memory, attention and decision processes*	Having access to checklists and training material to use as a reminder	Insufficient consultations to maintain up-to-date knowledge
	Having checklists close to the nonprescription sildenafil box as a prompt	Salient events distracting from following the points of the checklist
	Always asking questions in the same order	Pharmacists being uncertain of the need for sildenafil or needing to check contra-indications before supply
*Environmental context and resources*	Training (for pharmacist and for staff)	Not having a medical history of the client
	Having a checklist as a guideline during the consultation	Experiencing negative salient events during requests
	Presence of a private consultation room or area	Low public awareness of the availability of sildenafil on prescription
	More than one pharmacist and/or a male pharmacist working in the pharmacy	Advertisement that raises the expectation that a consultation is not necessary
	Accessibility of pharmacists and pharmacies	Men who did not know which medicines they use
	Information leaflets for patients *(potential facilitator)*	National culture of being reluctant to talk about sex
	Self-care being promoted by government policy	No anonymity in a rural pharmacy *(potential barrier)*
	Sharing experiences between staff members and staff understanding the pharmacists’ role in nonprescription sildenafil supply	A socio-economic deprived area in which potential clients cannot afford the high price of OTC sildenafil
*Social influences*	Positive reactions about nonprescription sildenafil from other colleagues and men returning for re-supply	Patients not understanding why pharmacist needs to ask questions about all OTC medicine
		Fear of offending older or religious persons by actively promoting the service
		Having to ask intimate questions to someone who is known to the pharmacist *(potential barrier)*
*Behavioral regulation*	Having a checklist as a memory aid	
	Using the checklist to record information about a client	
	Staff awareness of the pharmacists’ role in nonprescription sildenafil supply	

ED = erectile dysfunction; GP = general practitioner; OTC = over-the-counter; TDF = Theoretical Domains Framework.

*“The CPD* [Continuing Professional Development; training from a national or regional organisation] *gave me the knowledge about the condition and also about interactions that could possible happen with certain drugs that they may be taking but whether all that helped me with regard to interviewing somebody I don't know it just gave me the background knowledge, a lot of it is how you deal with the patient because it is safe”* (PH1 (F))

Some pharmacists had also ensured their staff had undergone the training so that they could help where possible, eg, asking a client if they had the resupply slip and if there were any changes in their health (‘Environmental context and resources’). More pharmacists mentioned ‘informed staff’ as a facilitator, although they generally referred to this as a safety measure (“Behavioural regulation”).

*“All the staff know that it has to go through the pharmacist for the Viagra consultation although sometimes the pre-reg* [the pharmacist in his or her pre-registration year] *would do some of the preliminary questions like have you had it before and if they have the slip they will always refer to me for the final sale”* (PH10 (M))

The manufacturer's checklist highlighted the restrictions for supply, with clear limits for the pharmacists’ role in the supply (“Social/Professional role and identity”). The checklist was used to explain the need for a consultation with a pharmacist, to guide the consultation, as confirmation that everything was discussed or to prove that consultations had taken place (“Memory, attention and decision processes” and “Behavioural regulation”). The checklist was often kept close to the nonprescription sildenafil boxes as a reminder to take the checklist to the consultation room. As another check, some pharmacists always followed the same order of consultation points (“Memory, attention and decision processes”).

The consultation room or private area was already available for other services, due to the importance of self-care in government policy (“Environmental context and resources”). Pharmacist and pharmacy accessibility, having more than 1 pharmacist working at a time and having a male pharmacist working in the pharmacy were considered facilitating resources to provide OTC sildenafil (“Environmental context and resources”).

*“I do know that other pharmacies have more than one pharmacist so yeah, it would be a lot more manageable then”* (PH8 (F))

Another facilitator that was consistently reported by the pharmacists was ‘experience’, leading to confidence, which in turn had helped them understand the situation of the client and to adapt their communication and negotiation skills to make the individual feel comfortable (“Beliefs about capabilities,” “Knowledge,” and “Skills”). Pharmacists were generally more confident about consultations in which sufficient information was passed on about the individual's health so that they could make either a safe supply or an appropriate referral to the GP (“Beliefs about capabilities”).

*“I feel better when somebody can relate to me their circumstances* [medical history]*… it's a bit safer whereas some other people are very vague about their health history”* (PH4 (M))

Experience with a successful consultation also indicated that the service was working, along with increased sales of OTC sildenafil, low numbers of inappropriate requests, positive reactions from other healthcare providers and clients returning for resupply (“Optimism,” “Reinforcement,” and “Social influences”). These experiences were considered rewarding, as well as the perception that pharmacists may have helped relationships by overcoming a taboo problem (“Reinforcement”).

*“People may have been nervous about coming in the first time, and now they come in and they have a big smile, and they just hand in the slip, and you just have a conversation, they say ‘no changes’, and they're just smiling, they're happy with what they've got”* (PH7 (F))

Most pharmacists considered themselves part of the changing pharmacy profession and showed commitment to professional advancement (“Social/professional role and identity,” “Intentions,” and “Goals”). These pharmacists believed that the provision of OTC sildenafil in the pharmacy would benefit the client, the pharmacy profession, the pharmacy and healthcare system (“Beliefs about consequences”).

*“It's easier for the patients to get the medication without having to try to get an appointment with their GP, it's better for the GP because it takes more of their plate because they are so overworked, it's good for pharmacy, it's another service and pharmacy is struggling at the minute so that's for pharmacy as a whole and it's good for the pharmacy itself because it's another service we can offer at the end of the day it's more profit”* (PH10 (M))

However, some pharmacists did not agree that OTC sildenafil would lessen the burden on the GP and healthcare system. From their perspective, the suggested health check during the consultation meant that men would still have to see their GP after the first supply. Moreover, men could buy OTC sildenafil and once they were confident about its effectiveness, could obtain it on prescription. In spite of these issues, these pharmacists still decided to provide the service because it could show their potential in providing such services (‘Social/professional role and identity).

*“It can be sort of researched and people can say okay that's done professionally that's done well and it works for the patient you know and it works for the health service let's try it again with something else”* (PH4 (M))

Some pharmacists also described how OTC sildenafil had become an opportunity to screen men for underlying conditions or lifestyle causes, to refer smokers with erectile dysfunction to the pharmacy's smoking cessation scheme, or to develop a professional relationship with men who had returned for re-supply (“Beliefs about consequences” and “Social/professional role and identity”).

*“If anything it is another screening nearly for the patient, maybe we can pick up something in the early stages, like of a disease, that we could then convince them that they need to see their GP and flag that up to their GP sooner rather than later, so we could have a really positive role in it”* (PH8 (F))

The most reported barrier was the lack of a clear medical history, including medications, from the client when he was not a regular visitor of the pharmacy (“Environmental context and resources”). This barrier made it difficult to check the supply restrictions. It was compounded by language barriers, by individuals who provided insufficient information or individuals who were at high risk for underlying diseases because they had not visited their GP in some time (“Beliefs about capabilities”).

*“They go ‘No. No. No. No. No’, and then you've got no information, and whether they have conditions or not, you're not going to know that”* (PH1 (F))

Related to this, many pharmacists reported the need for trust as a barrier: having to believe that a client had given truthful answers, would go to the GP when referred, and did not attempt to obtain a supply at another pharmacy (“Optimism” and “Beliefs about consequences”).

*“We can advise them to go to their GP after so many months for a review but whether they do that or not is another matter”* (PH6 (M))

When asked what would discourage pharmacists from providing nonprescription sildenafil, most reported inappropriate requests (eg, to enhance their performance), clients misusing the service (eg, lying about their medical history) and no indication that the service worked (eg, no resupply slips) (“Beliefs about consequences” and “Reinforcement”).

*“If they go through the consultation in one pharmacy and find out what not to say they can come in to another one and have all the right answers, and as far as I am concerned, I would think yes that's alright to sell, but then the patient might just be lying trough their teeth”* (PH10 (M))

Barriers that made the consultation more difficult were clients who did not have a clear need for sildenafil, several comorbidities and medications which required checking for eligibility, or uncertainty about ED causes (eg, alcoholism, depression) which would cause the pharmacist to hesitate to make a supply (“Beliefs about capabilities”).

*“I don't know is the honest answer, is it appropriate to sell it or not, because the patient's medication suggest that they've clearly been treated for a significant level of depression, it is not a low dose SSRI, it's a maximum dose of SSRI plus other medication, and I'm thinking in myself is it wrong to sell it in that context? Because they are stable in their situation as it is, and they would like to try that”* (PH4 (M))

Some pharmacists also considered it uncomfortable and not their responsibility to listen to men talking about their sex life during the consultation (“Social/professional role and identity”). Additionally, some pharmacists reported experiencing negative salient events such as drunk clients (“Environmental context and resources”) as possible distractions from the restrictions that had to be checked (“Memory, attention and decision processes”).

*“That he was drinking a* [alcoholic] *drink, it was challenging because it sort of belittled the whole medical side of what we're trying to do it … to get away from the proper medical side had been a bit sleazy”* (PH1 (F))

Pharmacists also reported that clients preferred a consultation with a pharmacist from their own gender, for example, males asking male pharmacists for OTC sildenafil and females asking female pharmacists for emergency hormonal contraception (“Social influences”).

*“I find that the men are more inclined to speak to me about it than any other members of staff. Sometimes I would have a female pharmacist working with me and they will request to speak to me about it”* (PH10 (M))

Another common issue that arose during interviews was the reluctance to talk about sex, which reflected Northern Irish culture (“Environmental context and resources”). One pharmacist would find it challenging to ask intimate questions to someone he knew and another pharmacist thought the service would not be possible in a rural pharmacy where communities are close-knit and anonymity might be difficult to maintain. Some pharmacists did not display the OTC sildenafil boxes in the pharmacy to avoid offending older or religious persons (“Social influences”). On the other hand, pharmacists were also not happy with advertisements outside the pharmacy, because those advertisements suggested that men could buy OTC sildenafil without consultation (“Environmental context and resources”).

*“Being where we are and having quite a lot of elderly population you wouldn't want to offend elderly people by actively advertising it* [OTC sildenafil]*”* (PH1 (F))

Pharmacists who worked in socio-economical deprived areas highlighted that the high cost of OTC sildenafil was unaffordable for many of their clients (“Environmental context and resources” and “Beliefs about consequences”). The pharmacist who had decided not to provide OTC sildenafil, also attributed the price as one of the main reasons why his clients did not ask for the service (“Intentions” and “Beliefs about consequences”). Low demand for the service was seen as a barrier by more pharmacists, largely because it then became difficult to maintain up-to-date knowledge (“Reinforcement” and “Memory, attention and decision processes”).

### Strategies to Overcome Barriers

Pharmacists had created strategies to overcome the recognized barriers. In the absence of an adequate medical history or with uncertainty if a client had attended a GP following a referral, pharmacists reported that they accepted that it was the clients’ responsibility to see their GP and to correctly report their medication and medical conditions they had (“Social/professional role and identity”).

*“You don't have their patient history so you just have to rely on the fact that they are answering you correctly and honestly”* (PH7 (F))

Some pharmacists addressed concerns about misuse through trust that the registration authorities would act if there were issues with OTC sildenafil. Others felt that the price was a sufficient barrier to minimise the misuse (“Optimism”).

*“The price is quite high so it cuts out the ‘I buy it for a mate and give it to a mate’ kind of thing...so no, there's no reason to stop it”* (PH1 (F))

For the consultation itself, experience seemed to be key in adopting the right attitude and methods.

*“You have to sit there and think about how am I going to ask that? What am I going to say?”*(PH4 (M))

Most pharmacists recalled the first consultations as somewhat difficult or uncomfortable. They had to find ways to make the patient feel at ease, to obtain the information that they needed from patients (“Beliefs about capabilities”).

*“It's a fine line between making it significantly difficult for someone who is quite embarrassed to start with and getting the proper amount of medical information to make sure that what they're taking is suitable for them”* (PH1 (F))

Pharmacists had learned how to always look friendly and confident, how to describe the technical terms with lay language and some would purposefully use words such as “sex” at the start of the consultation (“Skills”). To maintain a flow in the conversation, broad questions that covered many aspects of the checklist were asked at the start of the consultation.

*“As I got more used to it … instead of listing the specific drugs I would ask ‘what are you on’ and things like that and I've tried to be more casual about it so at the start I would have been very formal talking about sexual intercourse and now I find that that just makes the whole thing more awkward and more difficult for the patient”* (PH10 (M))

Several pharmacists reported that it was important to realize that it was normal to talk on a professional level about erectile function in a conversation with patients. They acknowledged their role in helping patients with their health, including sexual health (“Social/professional role and identity”).

*“You need to just get it into your head that you are there as a healthcare professional and you have to be professional and … maybe you might feel awkward or whatever but just don't allow for that because we're to help people and obviously if these persons came to look for this drug then they feel strong enough that they need some help so we shouldn't make it any worse for them you know we should support them”* (PH8 (F))

This patient-centered perspective was also used when talking about the price of OTC sildenafil in comparison to prescription sildenafil. If a client could not afford OTC sildenafil, the pharmacist would explain that it could be obtained on prescription for a significantly lower price (‘Environmental context and resources’).

*“If they look like £20 every couple of weeks is going be expensive then again I'd suggest them that they can possibly go see their GP and the GP do the private script for it”* (PH1 (F))

## DISCUSSION

This study adopted the TDF to explore the barriers and facilitators experienced by community pharmacists in providing OTC sildenafil. Most barriers and facilitators related to material resources (“Environmental context and resources”), professional identity (“Social/professional role and identity”) and building experience (“Beliefs about capabilities”) with the service. Men who requested OTC sildenafil were often embarrassed and their answers, sometimes short or vague, had to be believed on face value. To deal with these barriers, pharmacists had gained experience from the first consultations. Subsequently, they were able to conduct consultations more efficiently, were confident about their abilities, and knew how to elicit the necessary information. Notably, pharmacists were gratified for the perceived professional recognition, hoping it would lead to more pharmaceutical care services.

Some of the factors that the participants reported are common for any new pharmacy service, such as the need for training, time, and adequate resources. For the provision of OTC medication, a lack of medical history has been previously identified as a barrier for safe supply, and supply protocols and knowledge on the condition as facilitators.^34^ For pharmacy-based sexual health services, pharmacists had previously reported privacy issues, preferences about the pharmacist's gender and difficulties with sensitive questions as barriers, and a nonjudgmental appearance and improved job satisfaction as facilitators.^35^ We adopted the TDF to broaden our exploration of pharmacists’ experiences. In addition to the above-mentioned factors, we found that pharmacists who provided OTC sildenafil reported distinct aspects of the service they had to familiarize themselves with. These included the sensitive topic of ED, the anonymity of the service and clients who had no previous encounters with the health system. The latter aspect was also reported by the Medicines and Healthcare products Regulatory Agency (MHRA). They hoped that OTC sildenafil would encourage men to visit healthcare professionals who would be aware of potential underlying diseases causing ED and of the need for a health check.^36^

To our knowledge, this is the first study that investigated OTC sildenafil through the lens of behavior theory. In New Zealand (NZ), OTC sildenafil supply was also evaluated, focusing on pharmacists’ satisfaction with training and tools.^37^ The NZ model, available since 2014, includes notification of supply to the GP and stricter restrictions, for example, only for men aged 35-70 years who do not smoke and have no elevated blood pressure or diabetes. Consequently, the NZ model requires more contact with the GP about supply and more men who did not meet the requirements for such supply. Despite these differences, the NZ pharmacists reported similar experiences of changing the consultation's suggested structure to make it more efficient, feeling more confident with a checklist, appreciating the feeling of helping patients, and building new relationships with them. Additionally, some NZ pharmacists also cited “raising pharmacists’ profile” a benefit of the new service.^37^

As the first pharmacy-based sexual function service, OTC sildenafil broadens the scope of pharmacy-based care. In this way, the service aligns with the latest guideline from the National Institute for Health and Care Excellence (NICE) on the community pharmacy's role in public health.^19^ This guideline states that community pharmacies should gradually integrate into the existing healthcare pathways as “health and wellbeing hubs.” Important points from the NICE guideline are reflected in the manufacturer's suggestions on how to perform OTC sildenafil consultations, for example, raising awareness of potential underlying diseases causing ED and offering advice on lifestyle and medication causes of ED. However, we found that the pharmacists focused mainly on a safe supply rather than using the opportunity to refer to other services or offer lifestyle and medication advice. For example, only some pharmacists considered it an essential part of the first OTC sildenafil supply to suggest clients to see their GP for a health check within 6 months. In addition, sADRs as a potential ED cause were also rarely discussed, even though all pharmacists considered dealing with sADRs their professional responsibility. Pharmacists’ focus on safety has been identified before in a study about the most important factors in recommending OTC products to patients.^38^ They seldom considered the efficacy of an OTC product: a sale was made provided the OTC product was considered safe for the patient.

Additionally, the sensitive topic of ED might have made pharmacists even more reluctant to utilize the service for public health purposes. For example, pharmacists reasoned that they did not discuss sADRs in their pharmacies because patients did not wish for unsolicited information provision on sADRs. In another TDF-based study, pharmacists in Scotland were also interviewed about a sensitive topic: their care for patients with mental illness and addictions.^39^ In both cases, pharmacists were concerned about how these sensitive topics influenced their relationship with the patient. Pharmacists’ perspectives evolved through education and experience with patients. As patients were reluctant to talk with a pharmacist, trust had to be built. The pharmacists who provided care for patients with mental illness and addictions reported additional barriers to those experienced by pharmacists providing OTC sildenafil. There was a lack of consensus about where the pharmacists’ responsibility stopped and started, for example, some pharmacy teams disagreed if they should be listeners for patients with a mental illness if there were no medication-related reasons for the conversation.^39^ Furthermore, in another TDF-based study, pharmacists reported similar difficulties in providing pharmacy-based care to homeless persons.^40^ Pharmacists believed they could help homeless persons improve their health but considered the topic uncomfortable. Most did not feel it was their role to ask patients who might be homeless about their housing situation.^40^ Compared to providing care for the homeless or those with mental health issues, the sildenafil service was perceived as more manageable. This could be attributed to the support provided through training and a checklist. These tools helped pharmacists to gain experience and confidence with an erectile function service. Once these are achieved, it may be possible for pharmacists to expand the service to include a broader public health approach rather than a limited focus on sildenafil supply.

This study integrated theory in the design of the study by adhering to the guidance of the MRC Framework and adopting the TDF as a theoretical framework. The research group has considerable TDF experience, which is important in analysing and interpreting the findings.^29^ In addition, the topic guide was adapted from previous studies, informed by a broad literature search and piloted to refine the questions. Despite these strengths, several limitations should be noted. As with any qualitative study, the sample of participants may not be representative of the community pharmacist profession. However, the participants were purposefully sampled to reflect diversity. Although the sample size was small, data saturation was reached with 10 participants. Rapid data saturation is known to occur for studies with the aim to understand a behavior in a relatively homogenous group.^24^ It could be hypothesized that a young, female interviewer might receive different answers than an older, male interviewer. However, comparative research has shown that female and male interviewers received the same spontaneity and level of engagement of male interviewees.^41^ The relative inexperience and different native language of the researcher may have resulted in suboptimal probing for further information. Conversely, a foreign researcher may make fewer assumptions about pharmacy practice in a different country, thus reducing the risk on interviewer bias.^24^

This study provided insights into pharmacists’ perceptions on the provision of an erectile function service. Experience and support such as training and a checklist were needed for pharmacists to be confident about how they supplied OTC sildenafil. The service required trust in clients because of its anonymity. This study's findings also suggested that pharmacists were motivated and confident to provide care for a sensitive topic such as erectile function because the new service was accompanied with adequate support. However, pharmacists prioritized a safe supply over providing advice about lifestyle and medication causes of ED. Thus, to implement a sexual function service in the pharmacy, attention should be focused on the development of adequate support, the process of gaining experience and potential new skills, and once those are acquired, guiding the pharmacists to go beyond the safety aspects of the service. This new service is a small step in pharmacies becoming the “health and wellbeing hubs” as envisioned by NICE.^19^ The lessons learned from this study can be used to inform future research, policy and practice about the barriers and facilitators encountered when community pharmacists provide an erectile function service.

## STATEMENT OF AUTHORSHIP

Rineke Gordijn: Conceptualization, Methodology, Investigation, Writing – Original Draft; Martina Teichert: Conceptualization, Methodology, Writing – Original Draft; Melianthe P.J. Nicolai: Writing – Review & Editing; Henk W. Elzevier: Conceptualization, Writing – Review & Editing; Henk-Jan Guchelaar: Writing – Review & Editing; Carmel M. Hughes: Conceptualization, Methodology, Writing – Original Draft, Supervision.

## Figures and Tables

**Table:** 

TDF domain	Sub-theme	Summary of domain content
Knowledge	Knowledge	To supply sildenafil over the counter, pharmacists needed knowledge about the condition and the restrictions for over-the-counter supply. Several pharmacists mentioned not realizing how much interacted with or was contra-indicated for the use of sildenafil, especially alpha-blockers. Most also emphasized the knowledge on possible causes for erectile dysfunction, such as side effects of drugs like SSRIs, although some pharmacists mentioned not having the knowledge on all the possible causes to address that.
	Procedural knowledge	Pharmacists considered the different training opportunities and the checklist provided by the manufacturer adequate and necessary to ensure the safe and appropriate supply of over-the-counter sildenafil. Most mentioned they liked the checklist as a reminder or sometimes went back to the training to refresh their memory.
	Knowledge of task environment	Some pharmacists mentioned the need to understand the situation for the patient, who might have anxiety with the fact that they have erectile dysfunction or their lack of knowledge on possible causes of erectile dysfunction. Some were initially surprised with young, healthy men requesting nonprescription sildenafil.
Skills	Ability	All pharmacists considered themselves able to conduct the consultation. They had used different methods to improve the consultations, such as being happy and smiling to make the patient feel at ease, not using technical terms like Peyronie's disease and not shying away from using words like erection in the first sentences, talking with confidence to show their professionality, reassuring the patients that erectile dysfunction is a common condition and explaining the reason for the consultation and the referral to the GP.
	Interpersonal skills	To receive sufficient information from the patients, communication skills were needed to make the patient comfortable to talk. Pharmacists had to be empathic, discrete and understanding and should not allow themselves to be awkward. They should know how far they could go and, without seeming nosy or judgmental, give the patients the feeling that they could describe the issue and answer the questions. Especially for the referral to a GP, a pharmacist had to be tactful, have negotiation skills and be supportive, making sure to make the patient believed it was in the best interest of the patient to go to their GP and convince them to make an appointment.
	Skills development & Practice	Some pharmacists acquired their skills through training, for example with role plays or seeing good and bad examples. Some pharmacists took time to first think about how to ask the questions, but all agreed that experience from dealing with patients improved the consultation.
Social/ Professional role and identity	Professional identity	OTC sildenafil was seen as an extra service to provide, something to get recognition that a pharmacist is a professional. For most of the pharmacists, the service had already become an integrated part of the community pharmacy. Some pharmacists were frustrated with individuals who expected to be able to buy nonprescription sildenafil without consultation.
	Professional role	The pharmacists felt responsible to make sure that sildenafil would be suitable and safe for an individual. This professional responsibility was guarded if the legal obligations of any OTC drug and the specific guidelines for nonprescription sildenafil were followed. Some pharmacists mentioned other responsibilities as well: to educate patients on why the consultation takes place or why they should go see their GP, to suggest to individuals with less financial resources to get sildenafil on prescription and to give lifestyle advice as a possible way to improve erectile function. All pharmacists considered it their role to inform patients about sexual side effects of drugs (sADRs), although most would not discuss sADRs at the counter.
	Professional boundary	The restrictions on the dispensing of nonprescription sildenafil were in general clear and 1 pharmacist said that it worked smooth because it was not too strict. If there were doubts about the suitability, they would refer the individual to the GP, leaving the responsibility to make an appointment to the individual. Some pharmacists mentioned that they would consider talking about the individual's sex life as outside of their responsibility and comfort-zone.
	Professional confidence	Most pharmacists were confident that once pharmacists knew the rules, they could provide nonprescription sildenafil. However, for certain situations, some pharmacists doubted that restrictions and their professional judgement would be sufficient to assure safety and suitability. Some of the men requesting nonprescription sildenafil would not have been to a GP in a long time, and thus be unaware of potential underlying medication conditions. The use of sildenafil could then trigger a cardiovascular event. Some also doubted if they should provide sildenafil to female partners, men who would drink a lot of alcohol or to men who had psychological problems, even though the dispense would legally be allowed.
Beliefs about capabilities	Perceived behavioral control	Some pharmacists felt that providing nonprescription sildenafil could be difficult if they believed there was something not right but the individual had not given any reason not to provide it. Examples were when the individual did not seem willing to have a detailed consultation, only gave short or vague answers or when there was a language or cultural barrier. The easy consultations were patients who were happy to talk, and either had proof of good health or clearly named red flags as reason for referral. Other pharmacists felt that the provision of nonprescription sildenafil was not difficult at all because all the tools were there for pharmacists to do their job.
	Professional confidence	All pharmacists felt confident about how they provide nonprescription sildenafil. Two male pharmacists believed they were more confident to ask direct questions about erectile dysfunction than younger, female pharmacists. The 2 pharmacists who had not provided the service also said they would be confident, one of them only if he knew the individual and knew the cause of erectile dysfunction.
	Perceived competence	Most pharmacists mentioned experience as the source of their confidence, because of experience from their training, or learning from previous consultations. One pharmacist mentioned female pharmacists having to build their confidence for these consultations more than their male colleagues.
Optimism	Optimism	Increased sales, patients coming back for resupply and no complaints, inappropriate supplies or public criticism had made pharmacists optimistic that dispensing nonprescription sildenafil had positive outcomes for the individuals and for the healthcare system.
	Pessimism	All pharmacists had concerns about the level of trust needed for the service, because patients were unknown in the pharmacies and said they didn't have any medical history, because the patient could give the box to a friend or because some individuals had requested sildenafil for recreational use. Some pharmacists were also concerned that patients who were referred, might try again at another pharmacy or would not visit their GP. One pharmacist did not want to put the boxes in sight for the customers out of fear for abuse of the service. The high price for the box decreased their concerns somewhat.
Beliefs about consequences	Outcome expectancies	Most pharmacists hoped that having another service, nonprescription sildenafil, would show the general public what pharmacists can do for them and how pharmacists could expand their role even more. They expected that the high accessibility and the anonymity of the community pharmacy would help patients to get over their embarrassment to request sildenafil. In the end, it would help the individual to resume a happy relationship. Most pharmacists also believed that it would reduce time for GPs and costs for NHS and that it would improve public health because patients were referred for a health check or encouraged to stop lifestyle causes of erectile dysfunction such as smoking. However, some pharmacists believed that the men would still visit their GP and considered the high price of the over-the-counter variant vs generic sildenafil as a disadvantage.
	Consequents	The pharmacists felt that the service had uplifted the profession and most acknowledged small financial benefits from the sales of Viagra Connect®. Several pharmacists believed that the service had improved their skills and that is was better use of their skills. Because of the consultations, the confidence of 1 pharmacist had increased and she was happy to develop relationships with some of the returning clients. Although many were concerned about misuse of the new service, only 1 pharmacist was sure he had received inappropriate requests.
Reinforcement	Rewards, incentives	For some pharmacists, improved professional recognition was considered a reinforcement to provide nonprescription sildenafil. Others mostly looked at the demand for the service, only providing nonprescription sildenafil if many patients asked for it. Some managers also acknowledged the financial reward of a sale and getting more men into the pharmacy and other pharmacists believed that the bigger chains only provided it because of this financial reward. With the service up and running, reassuring reasons to keep providing the service were men returning for resupply, persons who are known to the pharmacist asking for the service, the feeling that you can help the relationship of this person and using the consultation as a reason for lifestyle advice.
	Punishment	Some pharmacists also named situations in which they would consider to stop the provision. Several pharmacist would be concerned it they did not see patients coming back for repeat sales or if they saw or heard of people misusing it. One pharmacist talked about looking out for negative feedback from patients or the authorities, such as adverse events appearing. Another pharmacist mentioned embarrassing occasions as reason for discouragement.
Intentions	Stability of intentions	Most pharmacists did not have to think for a long time if they wanted to provide an extra service, only 1 decided to wait to see if there would be a demand in his pharmacy and was surprised to see there was. This pharmacist said it was not about making sales, but about providing the service, and highlighted that he always reassured the patient that erectile dysfunction is common. In contrast, a pharmacist in the touristic center believed providing more services was financially necessary, because he wouldn't be able to survive on the funding from the NHS. All pharmacy owners considered it important to be part of progression, except for the pharmacist who did not provide the service. He did not see any reason to support Pfizer unless his own patients would ask for it. In his opinion, he always put the patient first and this service did not benefit the patient. He did agree with the other pharmacists that is important to be ready to provide it, most therefore underwent training before the launch of nonprescription sildenafil. One pharmacist was very strict about not dealing with women who request Viagra for their partner, another pharmacist was strict about not advertising the service because it might offend the elderly population.
	Stages of change	One pharmacist decided during the interview that he would want to talk about the cause of erectile dysfunction in his consultation and provide information about the condition. Another pharmacist had become more willing to talk about sexual problems with patients.
Goals	Goals (distal/proximal)	The pharmacy owners all considered providing OTC sildenafil as progression, a move forward that they had to be part of. Two pharmacists were strongly committed to promoting selfcare and saw promoting good public health as one of the main goals of the service. One of them had recently created an open ‘private area’ because of the direction in which pharmacy was going in his eyes. Two locums also said they would want to offer as many services as possible if they had a shop.
Memory, attention and decision processes	Memory	If there were no regular sales, it was difficult to keep everything in mind. Therefore, all pharmacists either felt that they could use a refreshing from the training or they went back every now and then to the literature. Some used the checklist as a reminder of what they would have to cover in the consultations and others kept the anonymously filled-in checklists, to have paperwork as their memory that supplies had been made and why.
	Attention& Attention control	The checklist was also considered useful to keep attentive to the most important consultation points. Some mentioned keeping the checklists close to the boxes as a prompt to use it. Most would always follow the same order of points to control themselves. Salient events such as drunk men requesting the service, made it difficult to control the attention towards these points. Some mentioned informing the pharmacy team that it is a pharmacist-led service as a way of control. Two pharmacists mentioned being more attentive, one of the additional advice and the other of the impact erectile dysfunction can have on someone's life.
	Decision making	Although the decision to supply or not supply was considered straightforward looking at the points of the checklist, there were some situations in which it was more difficult, for example examining if there is a genuine need for sildenafil or having many comedication and comorbidities which are not contra-indicated.
Environmental context and resources	Resources/material resources	The training and the checklist were useful resources to provide the service, as well as training for staff, a private area, more than 1 pharmacist present, a male pharmacist present and long opening hours. The checklist was considered concise enough, easy to take with you in the consultation and the addition of the slip to give to the patient was also praised. Two pharmacists worked with a private area other than a consultation room and had good experience with this. Some pharmacists had the Viagra Connect® boxes in sight at the counter, but other believed it would be better not to promote the service. Some believed having a busy pharmacy could be an issue, but none had had this experience. Both the financial reward as professional recognition were important. As improvements in resources, almost all mentioned having the medical history of the patient, some wished the training had focused more on interactions and communication skills, some mentioned that the additional advice did not stood out sufficiently, 1 pharmacist wished there was something that could be given to patients with official information and 1 locum mentioned sometimes running out of stock.
	Environmental stressors	In Northern Ireland, pharmacists often do not have the medical history of the patient available. Several pharmacists also mentioned not being confident that in the UK referrals in general would go where they were supposed to go. For OTC sildenafil this was particularly important because many men would be in the age group that may have blood pressure problems. Some pharmacy owners believed that they had to offer sufficient services because they would not be able to financially survive from only dispensing prescriptions in Northern Ireland. The investment of the government in selfcare had made 1 pharmacist change the interior of his pharmacy. One pharmacist worked as a locum at the border with the Republic of Ireland, where customers would cross the border for OTC medication because in the Republic of Ireland they would have to pay 50-60 euros to ask their doctor the same medication. This pharmacist, however, had not yet had a OTC sildenafil request in that pharmacy. Most pharmacists recognized that the checklist had to be exhaustive for legal purposes and the price high enough to not attract wrong customers, but some still felt that the price was too high for their population. Most pharmacists compared the price of OTC sildenafil to the free NHS prescriptions for sildenafil that persons with certain chronic conditions are entitled to, although the requests for OTC sildenafil were often men who were not entitled to the free prescription. Two pharmacists mentioned that the public awareness of private prescriptions of sildenafil seemed to be lower since the start of OTC sildenafil. One pharmacist felt the margin suggested by Pfizer was under valuating the input of the pharmacist. Several pharmacists said the service was heavily advertised on television, with some dissatisfied about how it was advertised as if no consultation would be necessary. The OTC sildenafil service was considered more appropriate for city centers than rural areas where the service would not be anonymous.
	Person x environment interaction	The national culture of being reluctant to talk about sex made the service more difficult, as well as a language barrier with foreigners who also might have different expectations of pharmacy practice. One pharmacist had females asking if they could answer the questions for their partner because of the embarrassment around the topic. The men requesting OTC sildenafil were often from another area or unknown to the pharmacist, with some exceptions. One pharmacist started providing OTC sildenafil because he had patients inquiring about it. Another pharmacist mentioned that patients were occasionally surprised to find out sildenafil could be bought over-the-counter. Some patients didn't know what medication they were taking, gave vague answers, were unwilling to answer or unwilling to pay, making the consultation more difficult. One pharmacist was frustrated with the public lack of understanding why questions would be asked before the supply of OTC medication. The same pharmacist considered sharing experiences between staff members as crucial to provide the service, whereas others mentioned understanding between staff and pharmacist about their role.
	Salient events/critical incidents	The pharmacists who had provided OTC sildenafil all had experienced salient events, requests that were unusual. Some salient events were about the indication: males with psychological causes for erectile dysfunction, men who were drunk or were known alcoholics, requests for recreational use or enhancement or requests from younger persons who seemed perfectly healthy. In some instances, the men requesting OTC sildenafil got upset about being referred did not take the pharmacist or consultation seriously, did not understand the pharmacist because of language barriers or were obviously lying to be able to buy sildenafil. One female pharmacist had females asking for OTC sildenafil for their partner and another pharmacist had a couple coming in together. One pharmacist did not sell OTC sildenafil because of bad experience with the manufacturer. Another pharmacist had more positive salient events: men who requested OTC sildenafil coming back to start the smoking cessation scheme.
Social influence	Social norms and social pressure	Some pharmacists mentioned expectations from the public that they felt were not aligned with how the service worked, for example that it was advertised on television that you could purchase nonprescription sildenafil in every pharmacy and without knowing somebody. On the other hand, advertisement also helped for common support for the product. Most pharmacists did not actively advertise for nonprescription sildenafil, out of fear for offending elderly or religious people. It was also considered socially unacceptable to inform about sexual adverse drug reactions (sADRs) at the counter. Some pharmacists also felt that their opinion was influenced by their own pharmacy staff, by other pharmacists who they were friends with, GP practices, the men requesting OTC sildenafil and for the locums, their managers. One pharmacist mentioned that he would be more awkward asking intimate questions to someone he knew.
Behavioral regulation	Self-monitoring	Pharmacists monitored themselves by following the checklist, some always in the same order, others by checking the points at the end. A few pharmacists also kept the checklist to have paperwork of the anonymous service. All mentioned that it was important that all staff was made aware that only pharmacists could do the consultation, often feeling more in control when they did the whole process themselves. Two pharmacists also had the checklist next to the boxes with sildenafil, to remember themselves to use the checklist.
